# Simulation Study of BPPV Fatigability

**DOI:** 10.3389/fneur.2022.874699

**Published:** 2022-05-06

**Authors:** Xiaokai Yang, Lidan Gao

**Affiliations:** Neurology Department, Third Affiliated Hospital of Shanghai University, Wenzhou Third Clinical Institute Affiliated to Wenzhou Medical University, Wenzhou People's Hospital, Wenzhou, China

**Keywords:** BPPV, diagnosis, physical engine, simulation analysis, otolith, fatigability

## Abstract

To analyze the mechanism and clinical significance of Benign paroxysmal positional vertigo (BPPV) fatigability and discuss how to eliminate BPPV fatigability. A physical simulation model of BPPV was developed to observe the effect of the Dix-Hallpike test on otolith location and explore strategies to eliminate fatigability. Dix-Hallpike test can keep the otoliths in the lower arm of the posterior semicircular canal away from the ampulla. When the head is tilted 30° forward, the otolith slides to the lower arm near the ampulla, which is sufficient to ensure that the starting position of the otolith is consistent when the Dix-Hallpike test is repeated. When the head is tilted 60° forward, the otolith can enter the ampulla and reach the bottom of the crista ampullaris, which leads to long latency because the otolith sliding in the ampulla does not cause an obvious hydrodynamic effect during the Dix-Hallpike test. The otoliths located on the short arm side of the posterior semicircular canal will break away from the short arm side and enter the utricle when the head is tilted 120° forward. The stable and consistent nystagmus induced by the improved diagnostic test may be a more important feature of BPPV.

## 1. Introduction

Fatigability is considered an important characteristic of benign paroxysmal positional vertigo (BPPV); i.e., repeated Dix-Hallpike tests can reduce or even eliminate nystagmus. Barany reported the first case of BPPV, and the fatigue phenomenon of BPPV has since been described (1). When Dix and Hallpike introduced the Dix-Hallpike test in 1952, the fatigability phenomenon was further described: repeated Dix-Hallpike testing weakened nystagmus and reduced the duration. The Dix-Hallpike test was repeated 2-3 times, and the reaction was found to be completely eliminated (1). As Barany pointed out, the reaction can be triggered again only after a period of rest (1). The clinical significance and mechanism of fatigability are controversial. Nystagmus induced by the Dix-Hallpike test is mainly characterized by latency and transience, and fatigability is also considered a typical feature of BPPV. However, repeating the Dix-Hallpike test to confirm fatigability is not recommended because repeated testing will unnecessarily cause vertigo symptom recurrence, which may cause discomfort and interfere with the immediate bedside treatment of BPPV (2, 3). BPPV fatigability is generally believed to be caused by large otoliths scattering into small pieces in the semicircular canal due to repeated operations. However, previous studies do not support this mechanical explanation for fatigue in BPPV. Dispersed particles settling in the semicircular canal are considered to lead to a larger cupula offset than a single particle of the same mass (4–6). Boselli's model can explain the finite size of particles and their hydrodynamic interactions (7). In 2014, Boselli quantified fatigability by measuring nystagmus intensity and analyzed fluid-particle dynamics based on a computer model of BPPV. Fatigability was believed to be due to repeated tests causing the distance between stones located in the lower arm of the posterior semicircular canal and the ampulla to increase (7). Boselli's explanation of BPPV fatigability is more convincing. Nystagmus does not always weaken with repeated diagnostic tests and can also be constant or even enhanced (7). In 2019, Imai studied and discussed the immediate effect and fatigability after the Epley maneuver. After 3 min of rest, the effect of the Epley maneuver was worse than that without rest. The direct effect of the Epley maneuver is considered to be caused by BPPV fatigability and not by the established therapeutic effect of the Epley maneuver itself (8). Fatigability can cause varying changes in nystagmus intensity under the same head position, which will interfere with the diagnosis and treatment of BPPV. The hypotheses are that the reason for fatigue is that the Dix-Hallpike test increases the distance between otoliths and the ampulla, and nystagmus will weaken or disappear when the diagnostic test is repeated. If the diagnostic test is improved such that the initial position of the otolith in the semicircular canal is consistent when performing the test, not only can BPPV fatigability be theoretically eliminated but nystagmus may also be consistent. This study attempts to further confirm the causes of fatigue and explore how to eliminate fatigue.

## 2. Materials and Methods

### 2.1. Establish BPPV Physical Simulation Model

#### 2.1.1. Image Data

One hundred patients with normal MRI findings of the inner ear and clear images of the semicircular canal and eyeball bottom were selected. The presence of local lesions that might affect the anatomical structure of the semicircular canals and the abnormal cranial structure was excluded. Using Siemens 1.5 T superconducting magnetic resonance system and standard head coil for inner ear examination. 3D constructive interference in steady state sequence (3D-CISS) (TR: 6.0 ms, TE: 2.7 ms, Fov: 135 mm × 180 mm, Marix: 256 × 192, Thickness: 0.7 mm) was performed (9).

#### 2.1.2. Segmentation for Semicircular Canal and Eyeball

We segmented the bilateral inner ear and eyeball models from MRI data with 3D slicer version 4.10.2 software (10). First, the Otsu method was used to segment the inner ear and eyeball models, and then the markers of segmentation results are expanded and extracted into the voxel model. Finally, the marching cubes function was used to transform the segmentation results into the surface model to make the surface of the models more smooth.

#### 2.1.3. Membrane Labyrinth Model in Standard Space Coordinate System

Based on 100 cases of the bilateral inner ear and eyeball models, the statistical shape model was generated, and the average model was derived as the standard model. According to the bifurcation point of the semicircular canal common foot and the lower edge of the eyeball as the horizontal plane, the standard three-dimensional spatial coordinate system was established (9). The bone labyrinth obtained by micro-CT data segmentation (11) was calibrated with the standard model, and then the three-dimensional spatial transformation of the membranous labyrinth was carried out to establish the spatial direction of the membranous labyrinth (12).

#### 2.1.4. Physical Engine

The BPPV physical simulation model is a powerful tool to visualize and understand otolith movement within the canals during the Dix-Hallpike test. Individual differences exist in the spatial direction of semicircular canal models; thus, simulation experiments using a single model may not be representative. The best strategy is to use different semicircular canal membrane models for BPPV simulations and conduct related experiments. However, because clinical CT and MRI cannot adequately show the membranous labyrinth structure, segmenting and obtaining a membranous labyrinth model is usually impossible. The membranous labyrinth model can be segmented according to MRI microscopy and micro CT imaging data but usually lacks spatial direction. An alternative method is to segment multiple bone semicircular canal models, establish a standard model, and then establish the spatial direction of the membranous labyrinth model by calibration, which can ensure the representativeness of the simulation model. In this study, we established a physical simulation model of BPPV based on the membranous labyrinth model, which was calibrated with the standard bone labyrinth model in the standard spatial coordinate system. The virtual simulation uses the bullet open source physics engine and Python language to control model rotation (12, 13). The hydrodynamic effect caused by otolith movement in the semicircular canal will cause deviation of the crista ampullaris and induce nystagmus. According to the principle of vestibular physiology, the nystagmus characteristics induced by otolith movement in different semicircular canals can be determined. However, the characteristics of nystagmus in this study are mainly based on the following generally accepted assumptions: when the otolith on the long arm side of the posterior semicircular canal is far from the ampulla, nystagmus induced by the Dix-Hallpike test is weakened; and otolith movement in the ampulla has no obvious hydrodynamic effect, and nystagmus is weak or absent.

### 2.2. The Effect of the Dix-Hallpike Test on Otolith Location

The long-arm side otoliths can be in the lowest position of the posterior semicircular canal in multiple places in the flat seated position. The short-arm side otoliths are located close to the ampulla in the flat seated position. Start the physics engine and take the otolith settlement position at the seated position as the initial position for the observation of the diagnostic test. The changes in otolith position after the Dix-Hallpike test were observed.

### 2.3. Explore Strategies to Eliminate Fatigability

The improved diagnostic test is intended to reduce the distance between the otolith in the lower arm of the posterior semicircular canal and the ampulla or to allow the otolith to reach the bottom of the crista ampullaris such that the initial position before otolith movement is consistent. When the head is tilted forward, the otolith in the lower arm of the posterior semicircular canal slides toward the ampulla. Starting from an upright sitting position with the head slightly tilted forward, the position change of the otolith under the action of gravity with an increasing tilt angle is observed, and the tilt angle is recorded when the otolith reaches key positions, including the long arm side entering the ampulla, the bottom of the crista ampullaris, and the utricle after exiting the short arm side.

## 3. Results

### 3.1. The Effect of the Dix-Hallpike Test on Otolith Location

**Figure 1:**
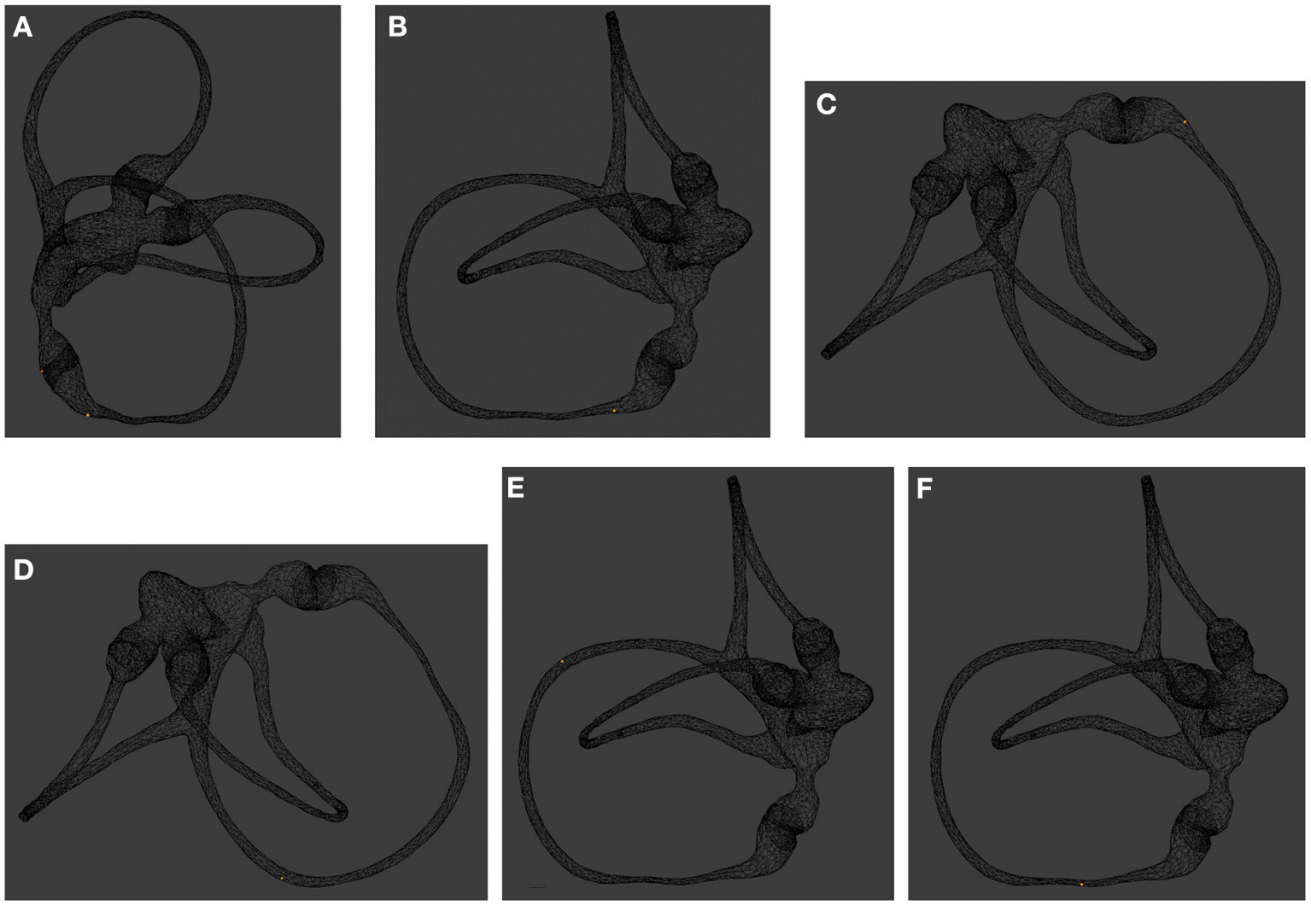
Otolith position changes in the Dix-Hallpike test. **(A)** Image taken with the patient sitting upright, back view. The otolith is located on the short arm side or near the ampulla of the long arm side of the right posterior semicircular canal. **(B)** Turn the head 45° to the 0 right. Lateral view. **(C,D)** Images taken with the patient lying supine with the right ear down and the head tilted back 30°. The otolith is initially located near the ampulla of the long arm side of the right posterior semicircular canal, moved away from the ampulla for a long distance. **(E,F)** The otolith is initially far away from the ampulla and moves toward the ampulla under the action of gravity, but it cannot migrate to the previous position and keep a further distance from the ampulla.

When the Dix-Hallpike test was performed with the patient in the supine position, the otolith moved a long distance away from the ampulla. However, when the patient assumed a sitting position, the otolith moved toward the ampulla under the action of gravity, but it could not reach its initial position and remained in the lower arm far from the ampulla (Figure 1).

### 3.2. Explore Strategies to Eliminate Fatigability

**Figure 2:**
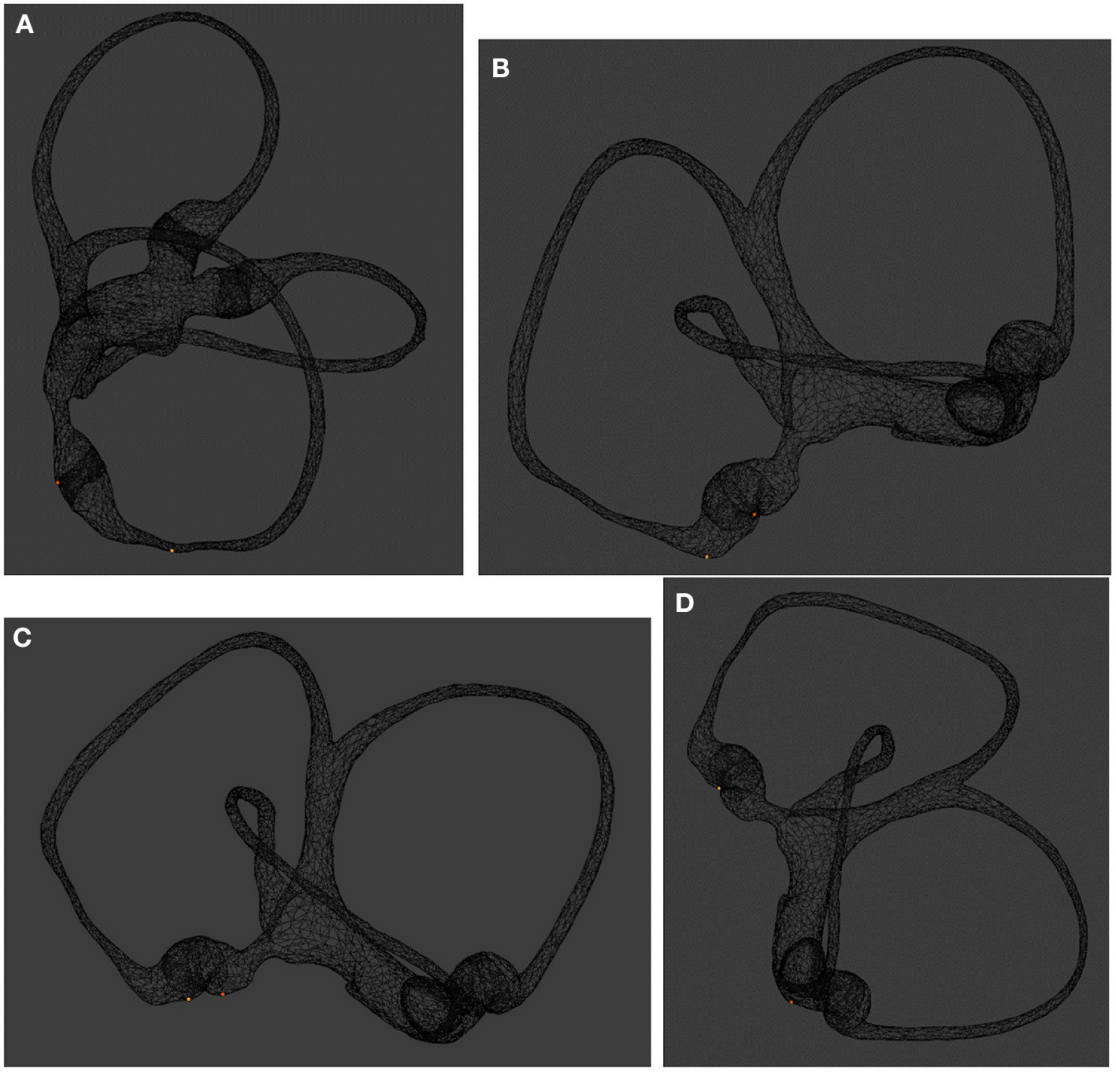
Effect of the bow maneuver on otolith position. **(A)** Image taken with the patient sitting upright, back view. The otoliths are located on the short arm and long arm sides of the posterior semicircular canal. **(B)** Image taken with the patient's head tilted forward 30°, lateral view; the otolith on the long-arm side moved closer to the ampulla. **(C)** Image taken with the patient's head tilted forward 60°, lateral view. The otolith enters the ampulla and reaches the bottom of the crista ampullaris. **(D)** Image taken with the patient's head tilted forward 120°, lateral view. The otolith on the short arm side of the posterior semicircular canal can exit the short arm and enter the utricle.

When the head is tilted 30° forward, the otolith slides to the lower arm near the ampulla, which is sufficient to ensure that the starting position of the otolith is consistent when the Dix-Hallpike test is repeated. When the head is tilted 60° forward, the otolith can enter the ampulla and reach the bottom of the crista ampullaris, which leads to long latency because the otolith sliding in the ampulla does not cause an obvious hydrodynamic effect during the Dix-Hallpike test. The otoliths located on the short arm side of the posterior semicircular canal will break away from the short arm side and enter the utricle when the head is tilted 120° forward. In conclusion, to achieve more characteristic nystagmus induced by the Dix-Hallpike test, the head is tilted forward at least 60° to cause the otolith to enter the ampulla and reach the bottom of the crista ampullaris (Figure 2). If the initial position of the otolith is consistent, then the otolith movement induced by the Dix-Hallpike test, its hydrodynamic effect, and the induced nystagmus should all be consistent, which is a reasonable assumption. When the Dix-Hallpike test is performed at this time, the starting position before movement of the otolith on the long arm side of the posterior semicircular canal, otolith movement, the hydrodynamic effect, and the induced nystagmus should all be consistent. Nystagmus will have the following obvious characteristics: a long latency, an upbeating rotatory pattern, a limited duration, a directional change upon assuming an upright sitting position, and no fatigability. Because the starting position of the otolith is consistent, the strength of nystagmus is mainly related to the nature of the otolith, including its size and the number of particles, which also have certain clinical significance. Different diagnostic methods induce different nystagmus characteristics. The traditional Dix-Hallpike test produces fatigue, which is considered one of the characteristics of BPPV. When the Dix-Hallpike test was modified by tilting the head to achieve a consistent initial position of the otolith, the fatigue phenomenon disappeared but recurrent and consistent nystagmus was induced in patients with BPPV.

## 4. Discussion

In most cases, experienced doctors can diagnose BPPV according to the typical corresponding medical history and nystagmus caused by changes in head position. However, when nystagmus is not typical, diagnosis can become complicated, especially for ordinary physicians. By observing both otolithic movements at different locations during the Dix-Hallpike test through virtual physical simulation experiments and nystagmus patterns, we found that six manifestations of nystagmus can be induced by the Dix-Hallpike test. If nystagmus does not follow the typical strong upbeat outward-torsional pattern, the specific location of the otolith is difficult to determine. According to the principle of vestibular physiology, the upbeat outward-torsional nystagmus induced by the Dix-Hallpike test may be due to excitatory stimulation of the ipsilateral posterior semicircular canal or inhibitory stimulation of the contralateral superior semicircular canal, which is caused by the otoliths in the contralateral utricle entering the superior semicircular canal through the common corus. It needs to be emphasized that inhibitory stimulation of the contralateral superior semicircular canal is not due to the original existence of BPPV of the superior semicircular canal, but that the otoliths in the contralateral utricle entering the superior semicircular canal through the common corus, which will almost certainly happen if there is free otolith in the contralateral utricle.

Fatigability is not necessary for the diagnosis of BPPV, and fatigability is not always present. The underlying cause of fatigue is a change in the position of the otolith as a result of the diagnostic test, which is related to the manipulation of the diagnostic test and the initial position of the otolith and does not reflect the essential features of BPPV disease. If the initial position of the otolith is away from the ampulla, fatigue does not occur, and even the opposite occurs with enhanced nystagmus. Different diagnostic test manipulations may also not induce fatigability. Based on the above reasons, repeating the Dix-Hallpike test to confirm fatigability is not recommended because repeated testing will unnecessarily cause vertigo symptom recurrence, which may cause discomfort and interfere with the immediate bedside treatment of BPPV. Diagnostic tests to induce vertigo and nystagmus are not sufficient for the diagnosis of BPPV, as other diseases can also induce vertigo and nystagmus when the head position changes. In many cases, it is difficult to exclude all other diseases, and the characteristics of nystagmus induced by diagnostic tests are consistent if the initial position of the otolith is consistent, due to the specificity of the pathogenesis of BPPV. Nystagmus will have the following obvious characteristics: a long latency, an upbeating rotatory pattern,a limited duration, and a directional change upon assuming an upright sitting position.There is good reason to believe that if the otolith initiation is consistent, repeated diagnostic tests showing consistent characteristic nystagmus manifestations, which are not present in other diseases, can be a sufficient condition for the diagnosis of BPPV. The fundamental purpose of our improved diagnostic test is to make the starting position of the otolith consistent, so that the induced nystagmus is more characteristic and can reflect the essential characteristics of BPPV disease, i.e., as long as the starting position of the otolith is consistent, then the repeat diagnostic test nystagmus performance is consistent and does not have fatigue. The purpose of our proposed head tilt of 60° is not only to eliminate fatigability, but more fundamentally for the sake of consistent otolith initiation; not for the sake of the diagnostic test repeatedly inducing vertigo symptoms, but for the sake of the diagnostic test repeatedly inducing consistent nystagmus manifestations, thus enhancing the sensitivity and specificity of the diagnostic test. Initialization of the position of otoliths by a 60° head tilt should require a certain resting period, to allow the otoliths to move to the starting point. The time taken for the otolith to slide into the ampulla varies with its initial position, and the physical simulation test results show that the longest distance takes 15 s. Considering that the size of the otolith may affect the settling time, the smaller the particle the longer the settling time, and keeping 30 s is appropriate.

Whether the otolith can be repositioned by tilting the head forward 120° can distinguish long arm side-type BPPV from short arm side-type BPPV (13). The temporal characteristics of short arm side-type BPPV latency should also be important indicators for identification, and further research and analyses based on video recordings of nystagmus are needed.

The initial steps of the Epley maneuver are consistent with the DIX-HALLPIKE test, so we attach great importance to the observation of nystagmus during repositioning and identify the initial steps of the Epley maneuver as a repeat of the DIX-HALLPIKE test. Only the repetition of the diagnostic test inducing consistent nystagmus performance is sufficient for the diagnosis of BPPV.

The clinical study shows that the modified Dix-Hallpike test (tilting the head forward 60°) had the same sensitivity and specificity as the classic Dix-Hallpike test, and there is no fatigue when repeating the Dix-Hallpike test (14).

Previous studies have analyzed nystagmus to locate otoliths through deep learning, but BPPV fatigability will have a considerable impact on the effect of deep learning (15). In the future, we need to collect nystagmus videos for further analysis and evaluate fatigability using nystagmus parameters (6). In conclusion, fatigability is related to changes in otolith position. The stable and consistent nystagmus induced by the improved diagnostic test may be a more important feature of BPPV.

## Data Availability Statement

The original contributions presented in the study are included in the article/Supplementary Material, further inquiries can be directed to the corresponding author/s.

## Ethics Statement

The studies involving human participants were reviewed and approved by the Ethics Committee of Wenzhou People's Hospital. Written informed consent for participation was not required for this study in accordance with the national legislation and the institutional requirements.

## Author Contributions

XY conceived, designed the experiment, and wrote the manuscript. XY and LG conducted the experiment. All authors read and approved the manuscript.

## Funding

This study was funded by the Natural Science Foundation of Zhejiang Province (Grant No. LSY19H090002) and Higher Education Teaching Reform Project of Wenzhou Medical University (Grant No. JG2020136).

## Conflict of Interest

The authors declare that the research was conducted in the absence of any commercial or financial relationships that could be construed as a potential conflict of interest.

## Publisher's Note

All claims expressed in this article are solely those of the authors and do not necessarily represent those of their affiliated organizations, or those of the publisher, the editors and the reviewers. Any product that may be evaluated in this article, or claim that may be made by its manufacturer, is not guaranteed or endorsed by the publisher.
