# Supplementary material for: Simulation Study of BPPV Fatigability
Source: Front Neurol. 2022 May 6;13:874699. doi: 10.3389/fneur.2022.874699 (PMC9121120; doi:10.3389/fneur.2022.874699)
Supplement: Supplementary file 1 [file Presentation_1.pptx]

## Slide 1
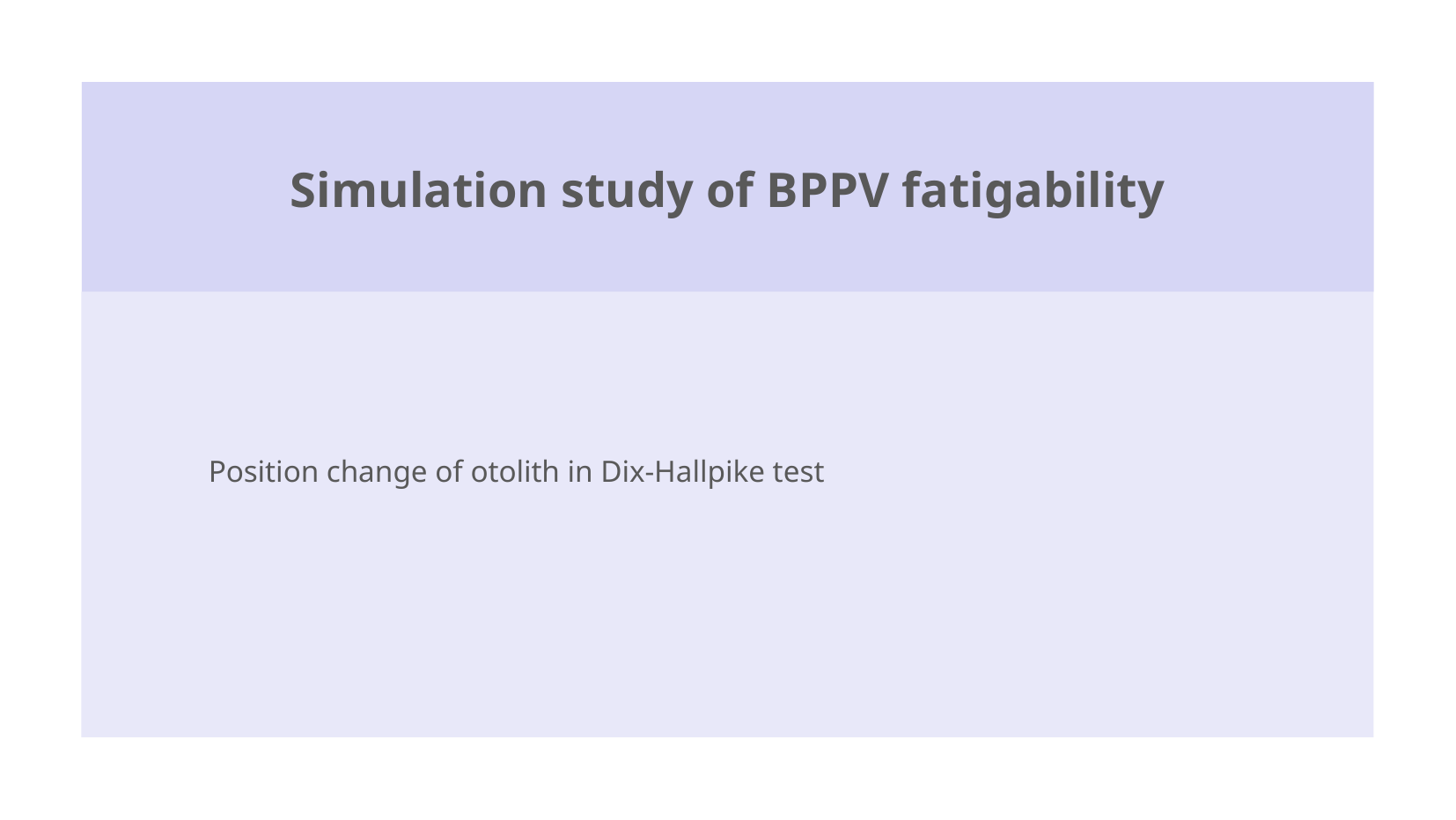

# Simulation study of BPPV fatigability
Position change of otolith in Dix-Hallpike test

## Slide 2
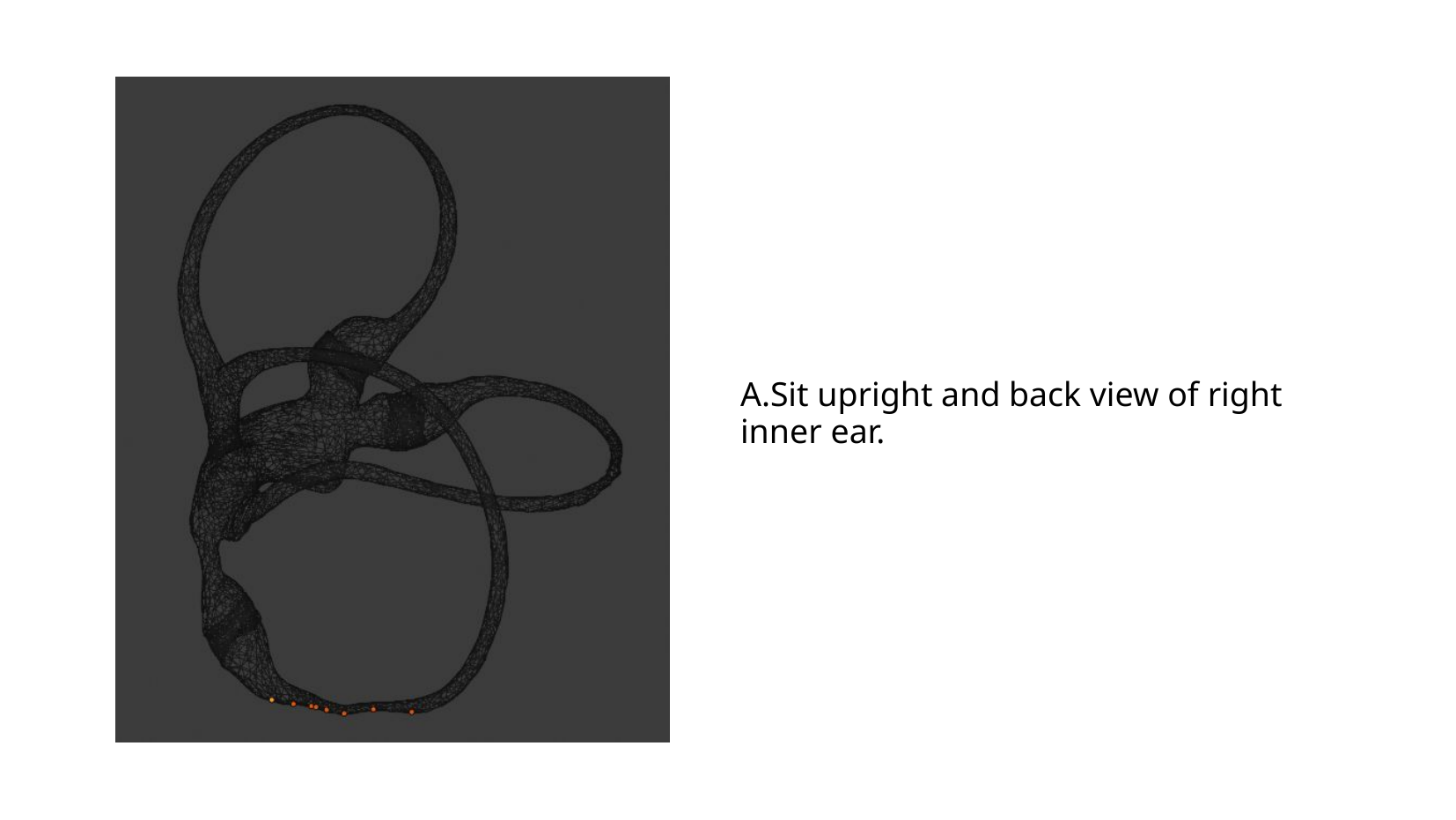

A.Sit upright and back view of right inner ear.

## Slide 3
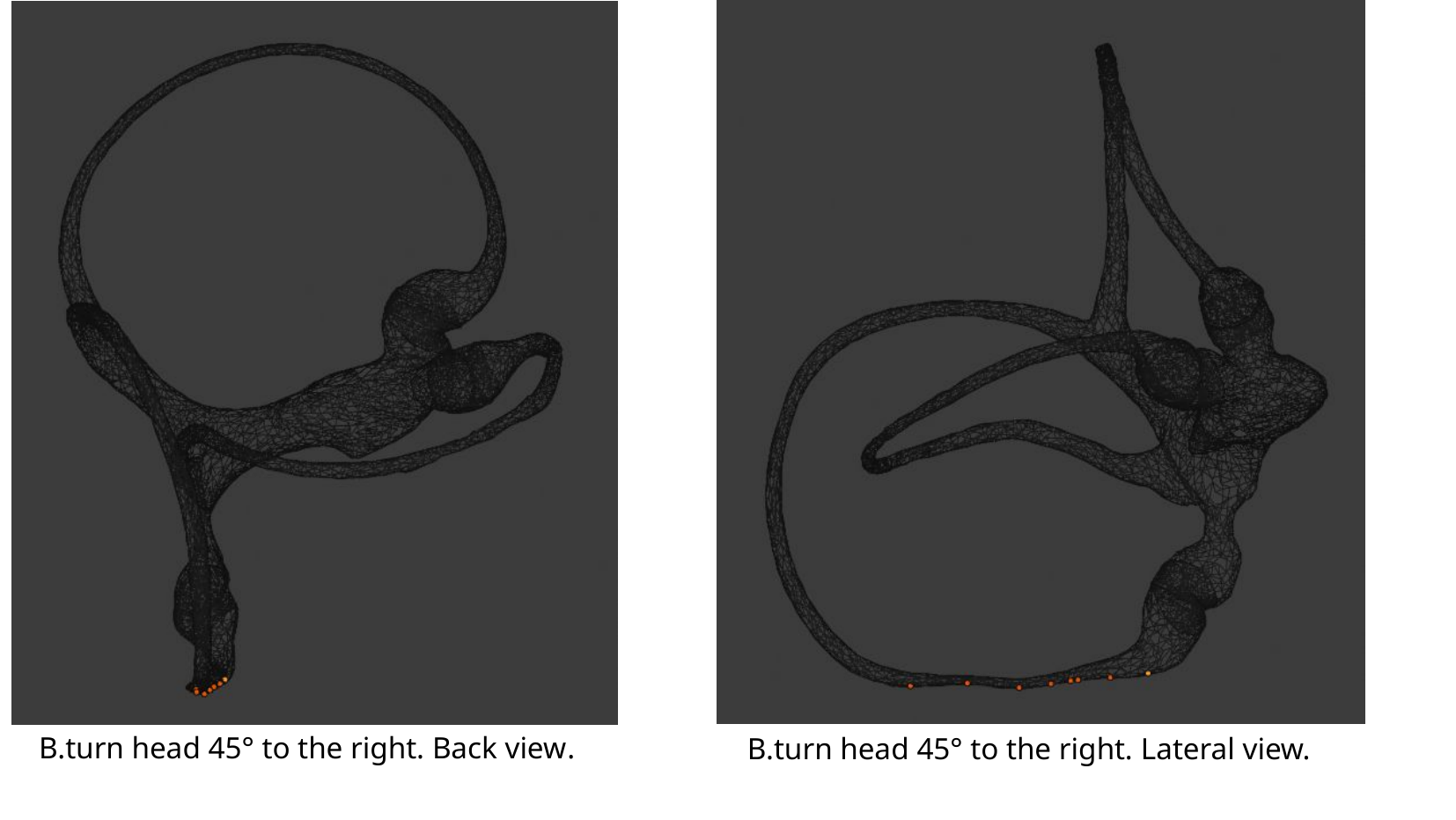

B.turn head 45° to the right. Back view.
B.turn head 45° to the right. Lateral view.

## Slide 4
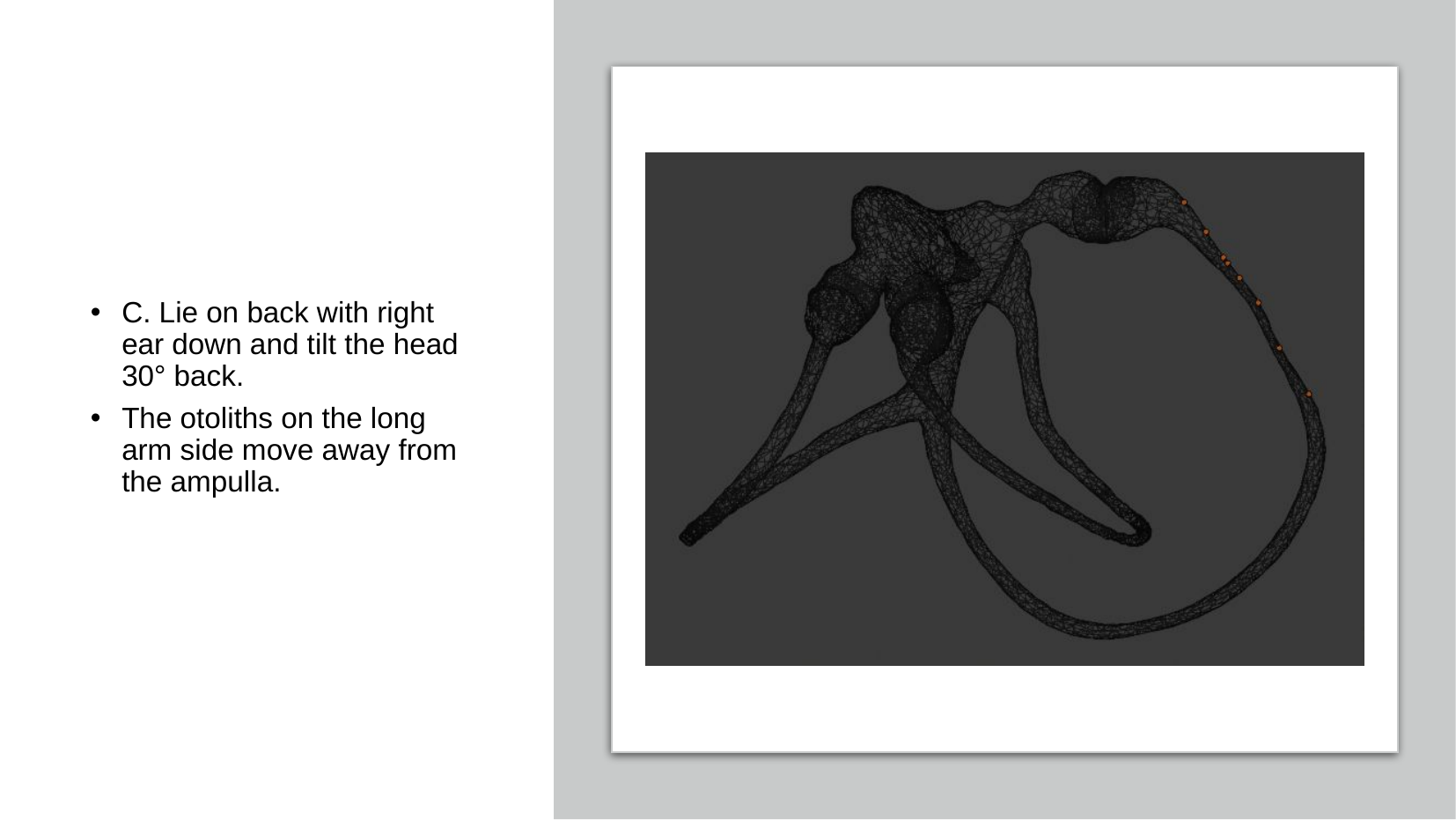

C. Lie on back with right ear down and tilt the head 30° back.
The otoliths on the long arm side move away from the ampulla.

## Slide 5
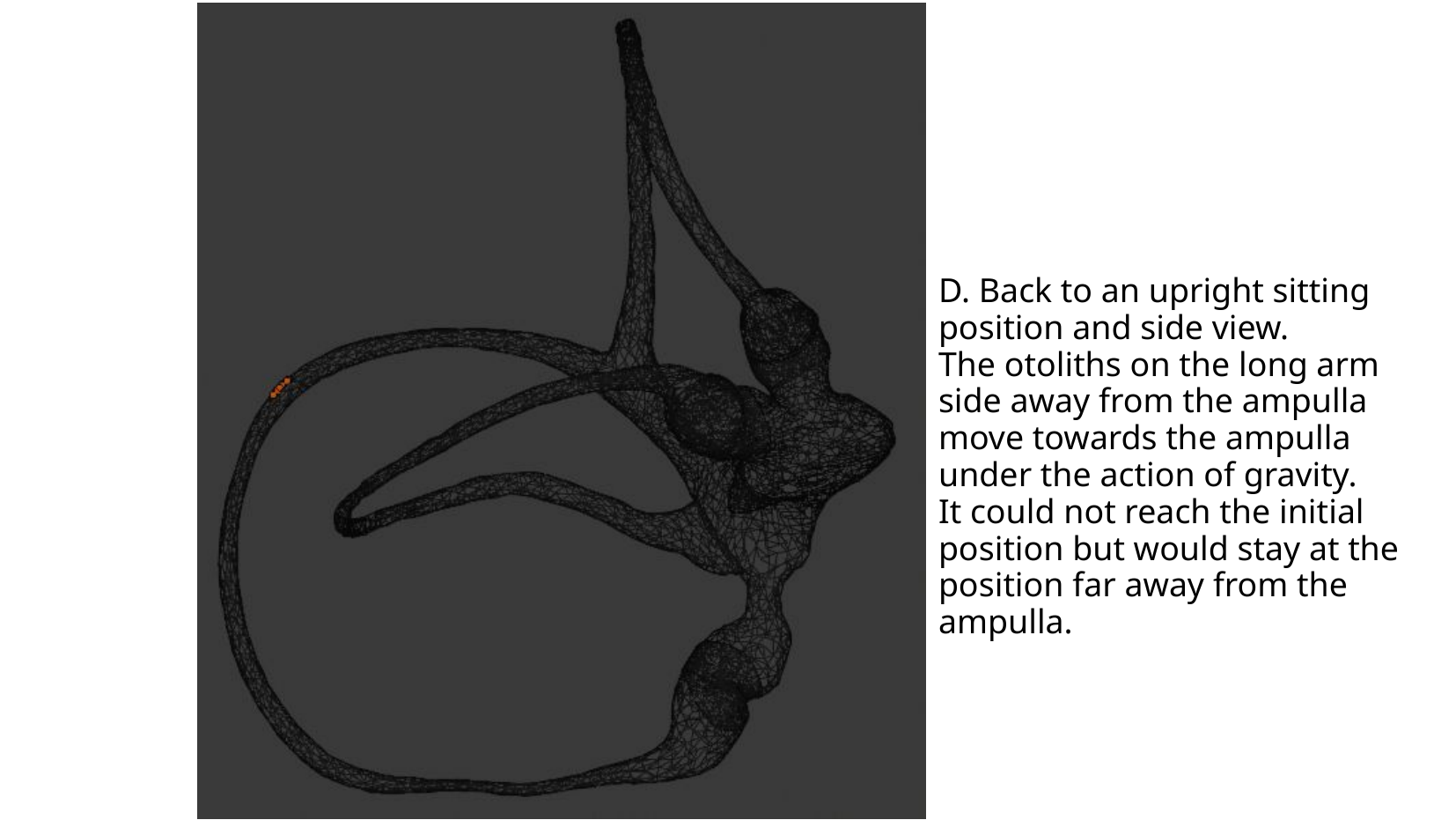

D. Back to an upright sitting position and side view.
The otoliths on the long arm side away from the ampulla move towards the ampulla under the action of gravity.
It could not reach the initial position but would stay at the position far away from the ampulla.

## Slide 6
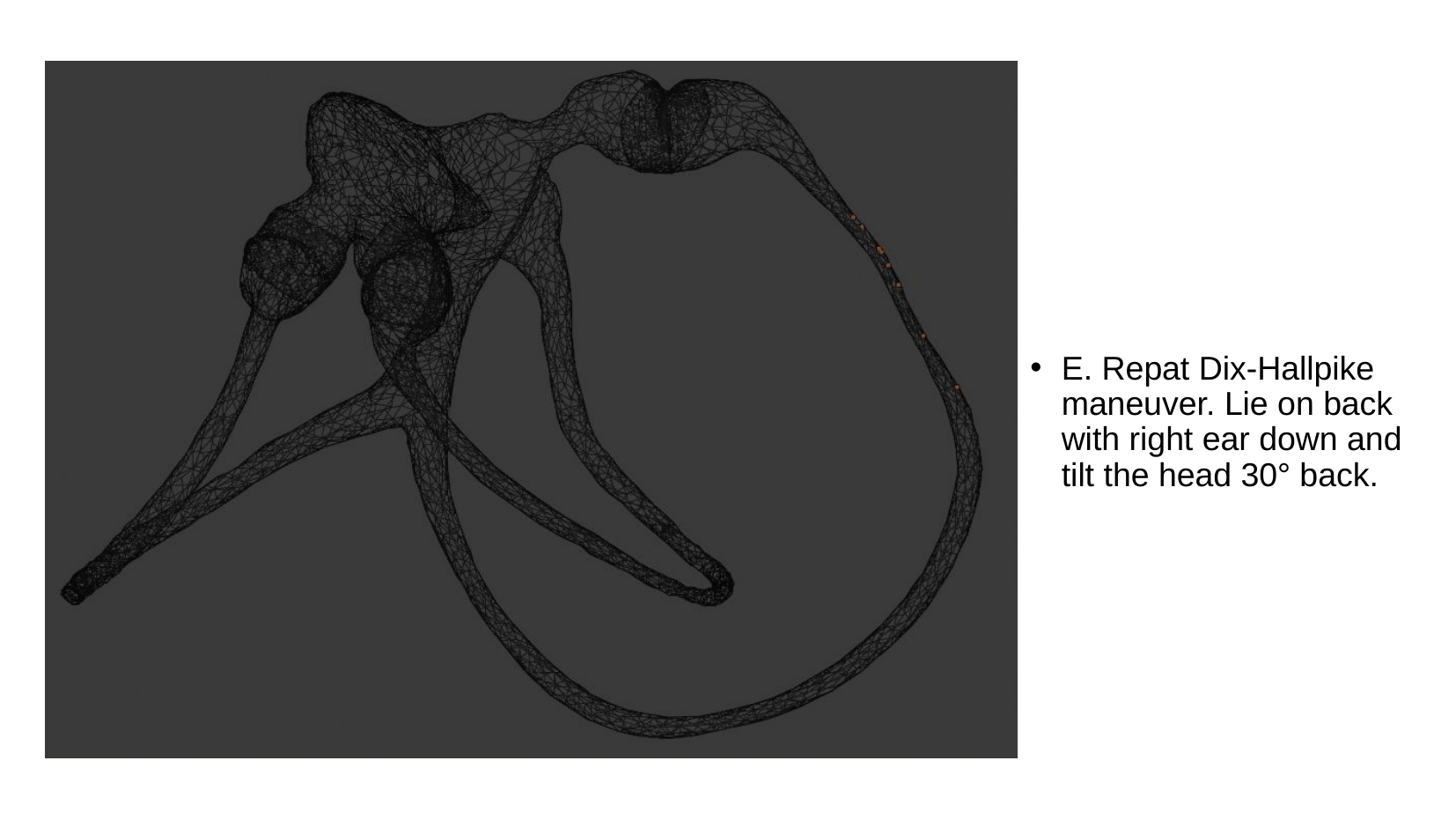

E. Repat Dix-Hallpike maneuver. Lie on back with right ear down and tilt the head 30° back.

## Slide 7
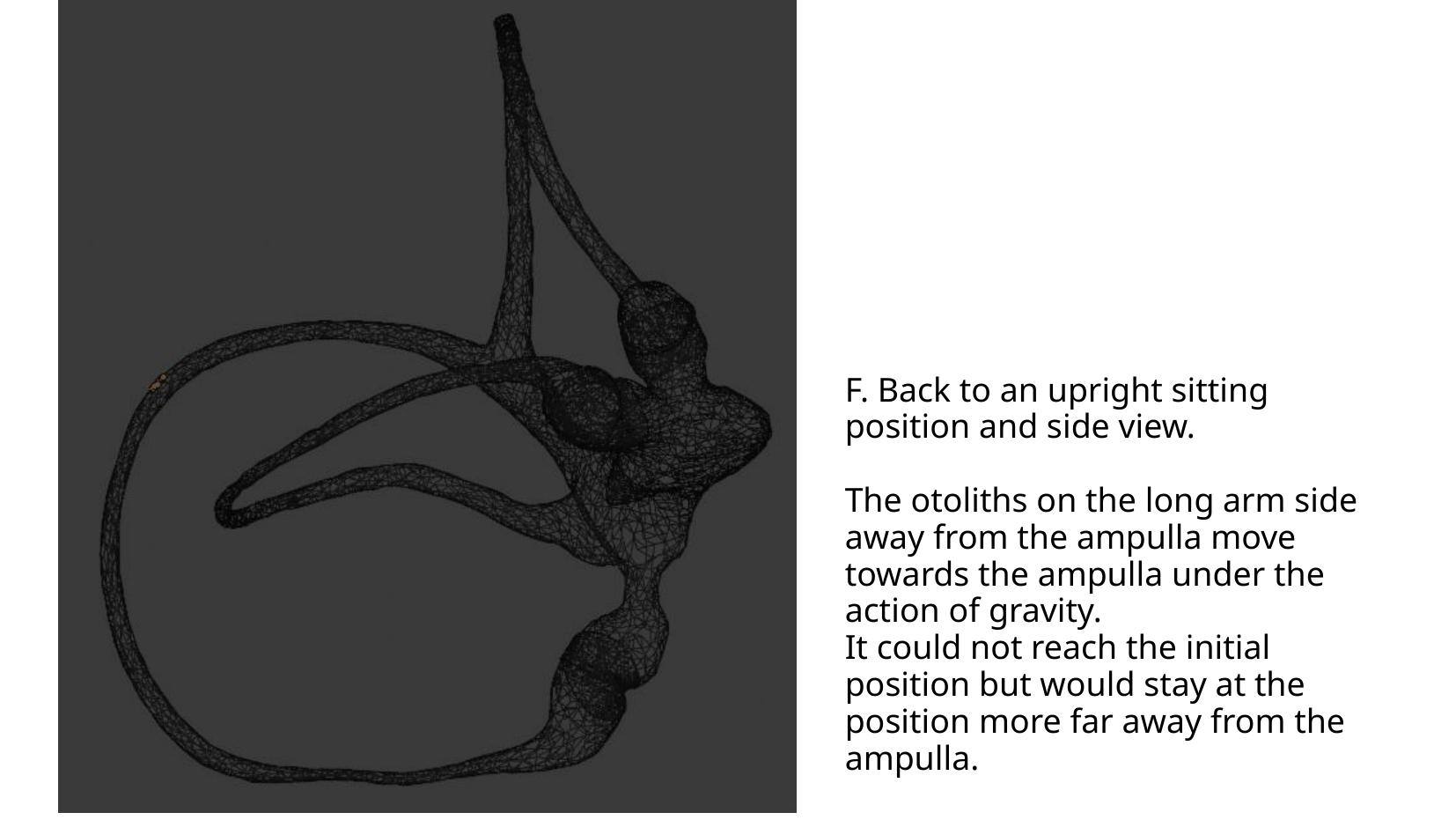

F. Back to an upright sitting position and side view.
The otoliths on the long arm side away from the ampulla move towards the ampulla under the action of gravity.
It could not reach the initial position but would stay at the position more far away from the ampulla.

## Slide 8
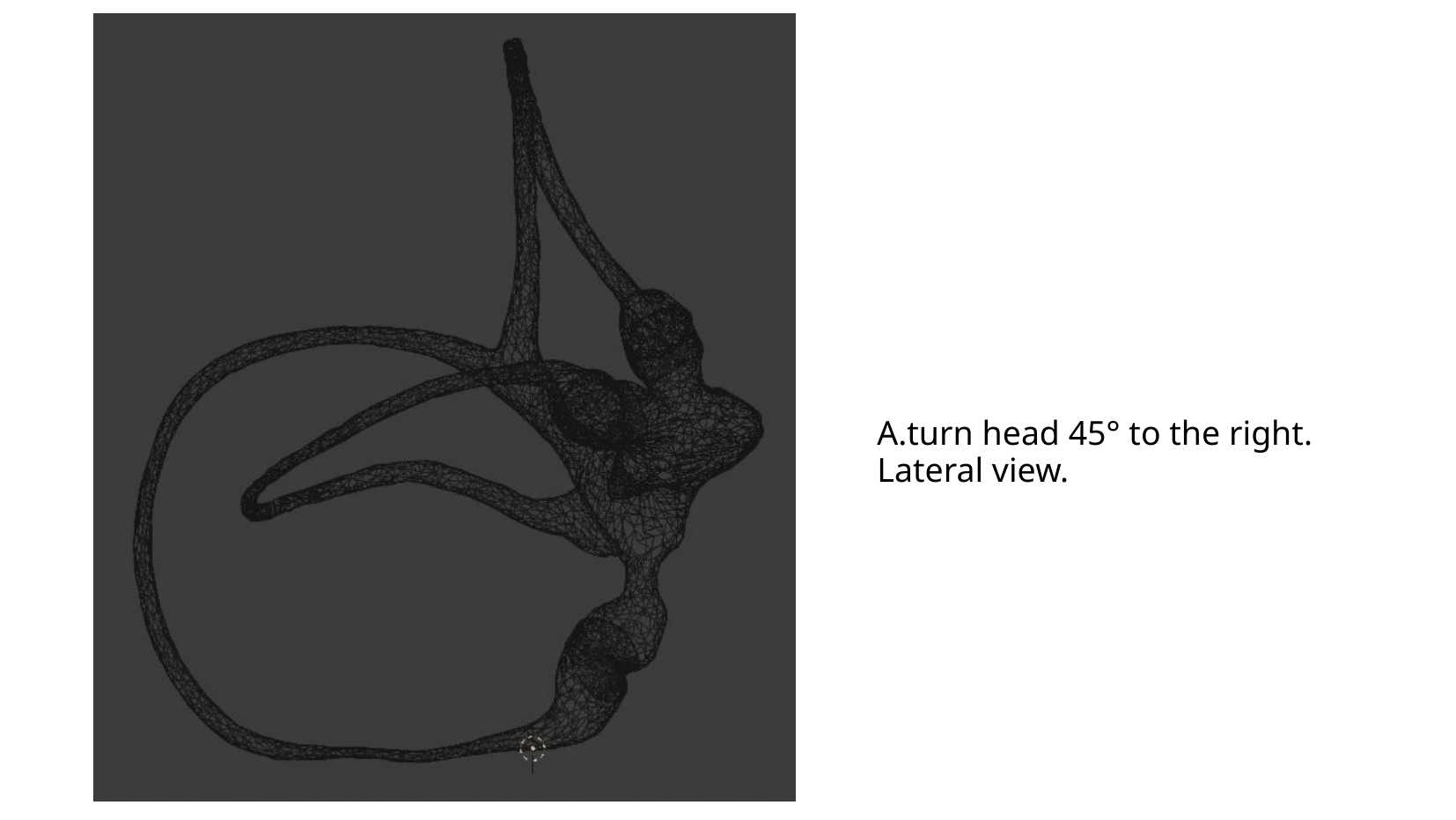

A.turn head 45° to the right. Lateral view.

## Slide 9
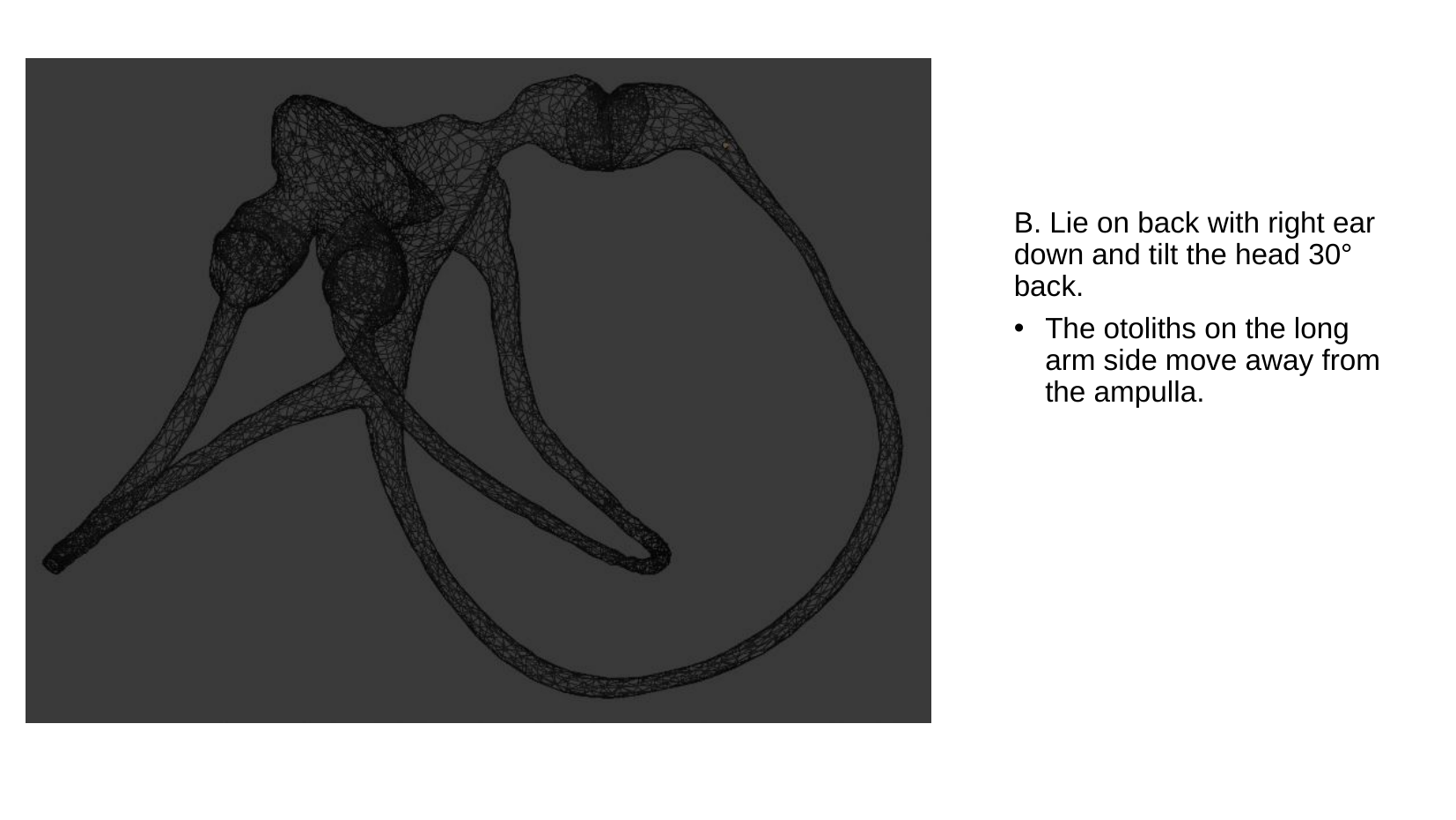

B. Lie on back with right ear down and tilt the head 30° back.
The otoliths on the long arm side move away from the ampulla.

## Slide 10
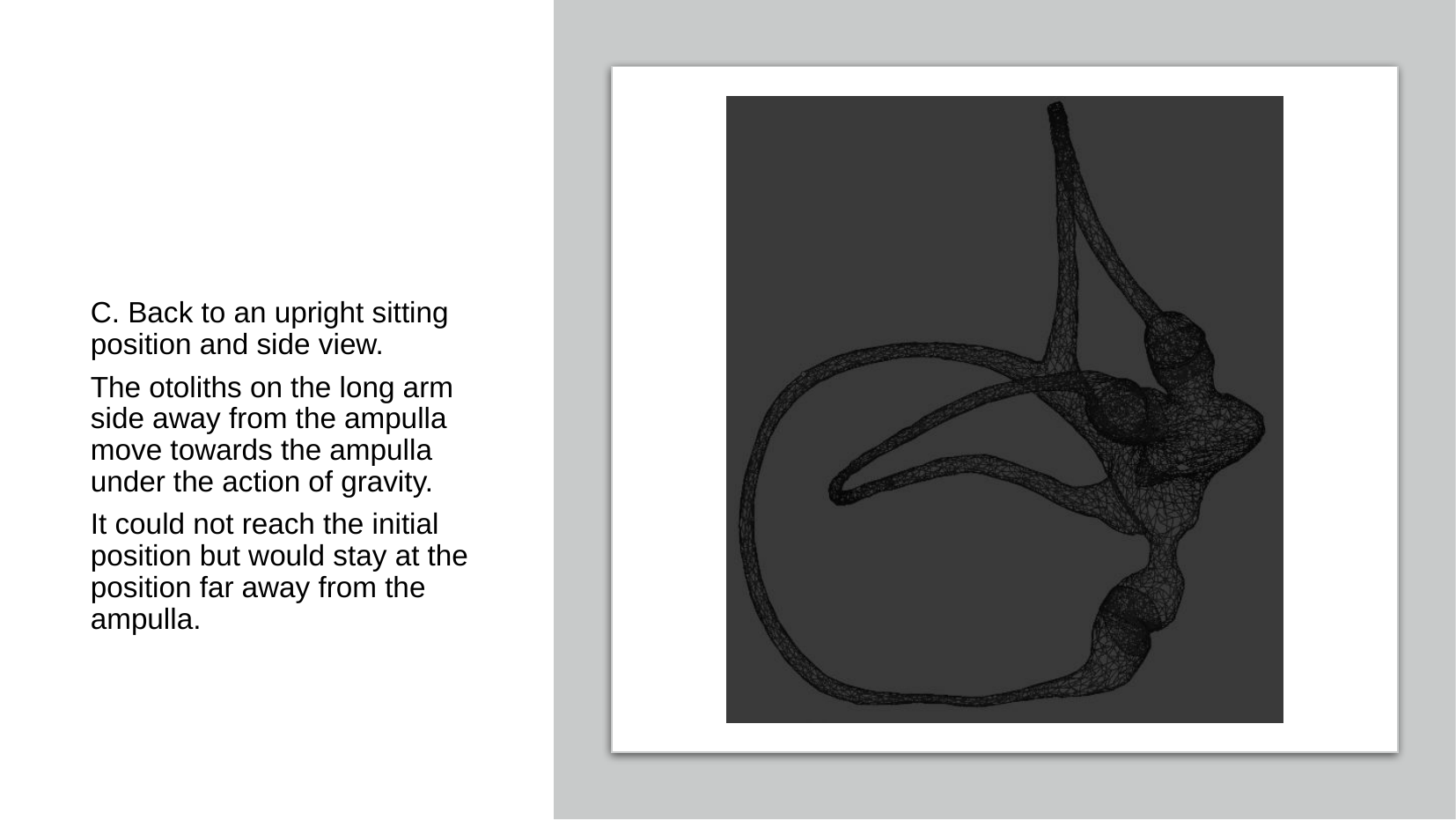

C. Back to an upright sitting position and side view.
The otoliths on the long arm side away from the ampulla move towards the ampulla under the action of gravity.
It could not reach the initial position but would stay at the position far away from the ampulla.
